# A Fast 3D Near Range Imaging Algorithm for a Scanning Sparse MIMO Array in the Millimeter Band

**DOI:** 10.3390/s20174701

**Published:** 2020-08-20

**Authors:** Feifan Wang, Bin Deng, Qi Yang, Hongqiang Wang, Ye Zhang

**Affiliations:** College of Electronic Science and Technology, National University of Defense Technology, Changsha 410073, China; feifanwang95@nudt.edu.cn (F.W.); dengbin@nudt.edu.cn (B.D.); wanghongqiang@nudt.edu.cn (H.W.); fighting_zy10@126.com (Y.Z.)

**Keywords:** MIMO array, near range imaging, range migration algorithm, synthetic aperture techniques, three-dimensional imaging

## Abstract

Millimeter-wave technology has been widely used in near range targets imaging scenarios, such as mechanical scanning and multiple input multiple output (MIMO) array imaging. Emerging scanning array regimes increase the need for fast-speed and high-quality imaging techniques, which, however, are often subject to specific array positions. Moreover, the relationship between array positions and the imaging performance is not clear, which leads to no uniform standard for array design. In this paper, a series of array configurations are designed to explore the impact of different array positions on the cross-range imaging performance. Meanwhile, a novel fast fully focused imaging algorithm with wavenumber domain properties is presented, which is not constrained by the positions of the transmitters and receivers. Simulation and experimental results show that, compared with a conventional algorithm, the proposed algorithm has a faster imaging speed under the same imagining quality. This study provides a feasible method for fast fully focused imaging in the case of location-constrained MIMO arrays, or partially damaged transceivers.

## 1. Introduction

With the development of transportation and expressage, the detection of hidden dangers becomes more and more urgent [1]. In terms of practical applications, there is a demand on detecting or imaging devices that has a high detection performance, and does not affect the normal flow of goods or people. Therefore, a fast imaging algorithm corresponding to a high-resolution 3D imaging system is significant for classifying weapons and explosives. Meanwhile, cost control in the detecting or imaging devices is very important to popularize them in the market. In order to reconstruct 3D image of targets, a 2D array or an equivalent 2D array must be used. The cost of building a plane array directly is usually expensive, which is made up of densely transceivers [2]. Therefore, through utilizing a scanning 1D array, an equivalent 2D array is widely adopted. The mechanical scanning and single input single output (1D-scanning-1D-SISO) system was firstly developed at the Pacific Northwest National Laboratory [3]. However, in order to prevent unwanted grating lobes, adjacent element interval of SISO array must be less than the order of a wavelength [4].Thus, the design of a 1D-scanning-1D-SISO configuration has become a challenge in a millimeter band. Furthermore, multiple input multiple output (MIMO) array techniques will contribute to extending array interval, enriching illumination patterns, suppressing side-lobe and increasing the dynamic range of the imaging performance. Thus, the mechanical scanning and multiple input multiple output (1D-scanning-1D-MIMO) array imaging technique has aroused extensive attention, which can exhibit both high imaging performance and low cost.

When imaging objects are illuminated by a series of millimeter wave (MMW) signals, many optically opaque objects appear transparent, which allows concealed dangers to be imaged. Meanwhile, MMW offers a non-ionizing radiation for the inspection of material [5]. During imaging, it is vital for the health of humans in the case of personnel screening. Due to a long array aperture paralleled with a wideband transmitted signal, the MMW imaging techniques based on 1D-scanning-1D-MIMO array can achieve fine spatial resolution, thereby forming high-quality 3D images. In recent years, researchers have made great strides in 1D-scanning-1D-MIMO array imaging techniques. Xiaodong Zhuge etc. proposed a high-resolution imaging system based on the combination of ultrawideband transmission, MIMO array, and synthetic aperture radar (SAR) [6].Then, David M. Sheen utilized sparse array techniques to achieve dense uniform sampling, which was suitable for high-resolution near-field imaging [7]. Frank Gumbmann etc. applied an extension of the sparse periodic array concept to optimize sparse array design, and proposed an optimized beamforming algorithm [8]. Bessem Baccouche etc. studied the influence of higher frequencies on 3D images and presented a novel highly sparse 3D terahertz imaging system to meet various kinds of requirements [9]. Jingkun Gao etc. presented two fast fully focused imaging algorithms, employing the spherical-wave decomposition formula [10]. The above-mentioned research studies are all involved in 1D-scanning-1D-MIMO array. Nevertheless, most of these studies focus on system design and array optimization. Although several fast imaging algorithms [10,11,12] are proposed, most of them are only suitable for specific array structure or cannot achieve fully-focused imaging, and there is not much quantitative analysis about the influence of array structure on the point spread function of MIMO array.

In this paper, an efficient fully focused imaging algorithm for 1D-scanning-1D-MIMO array is proposed, where both transmitters and receivers can be distributed for free in the 1D-MIMO array. For a practical application, considering the fixed interval between transmitter antenna array and receiver antenna array at the scanning direction, a compensation method is presented. The rest of this paper is as follows: In Section 2, the point spread function of MIMO array, spatial resolution and limitation of array interval are discussed. In Section 3, a fast algorithm and its formula derivation is presented, which is suitable for the case where both transmitters and receivers can be arbitrarily positioned. Meanwhile, the computational complexity is also analyzed in this section. In Section 4, simulation and experimental results are presented, respectively. The experiment system is introduced as well, which is equivalent to a 1D-scanning-1D-MIMO array. Meanwhile, the proposed algorithm is compared with typical and fresh imaging algorithm in imaging performance and time cost. In Section 5, the full work of this paper is summarized.

## 2. Array Design

The point spread function (PSF) is an important metric for 1D-MIMO array design, which describes the response of an imaging system to a point object. Ideally, it is a delta function, but in practice, a point may be mapped into a small area, the size of which will depend upon the aperture of the array used in the imaging system. [13]. As the PSF of 1D-MIMO array depends on the location distribution of transmitters and receivers, a direct expression of PSF can hardly be derived. However, it can be divided into two parts;

(1) $$PSF_{M}=PSF_{T}\cdot PSF_{R}$$

where $PSF_{M}$ is the PSF of 1D-MIMO array, $PSF_{T}$ is the PSF of transmitter array and $PSF_{R}$ is the PSF of receiver array. It means that just considering $PSF_{T}$ and $PSF_{R}$ respectively can form the PSF of 1D-MIMO array. Suppose $PSF_{S}$ is the PSF of 1D-SISO array based on transmitter array. Moreover, the only difference between $PSF_{T}$ and $PSF_{S}$ is that MMW of the former travels in one-way, while the latter’s MMW travels in two-way. Therefore, resolution and sampling criteria are discussed based on the 1D-SISO array.

### 2.1. Resolution

**Figure 1:**
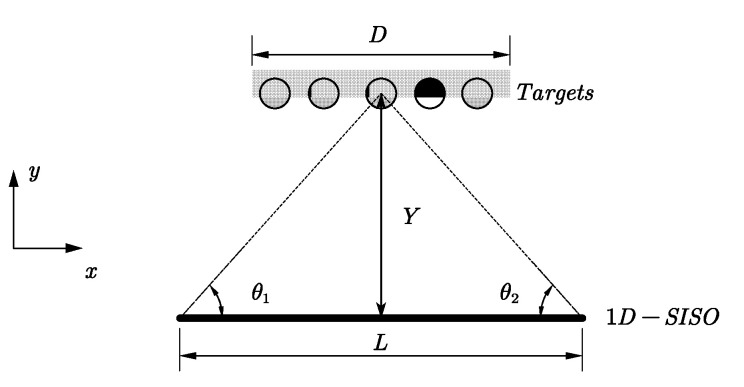
The observation scenario.

The geometry and coordinate axis setting of 1D-SISO array and a row of targets are given in Figure 1. The *x*-axis represents the dimension of the 1D-SISO array, and the *z*-axis represents the scanning dimension. The length of the array aperture is L. D denotes the distribution range of the targets in the *x* direction, and Y is the range distance from the targets to 1D-SISO array in the *y* direction.

In the following analysis, it is assumed that all targets can be illuminated by transmitters. Employing the conclusions of previous works [6], the down-range resolution $\delta_{y}$ is determined by the bandwidth B of the transmitted signal, and is given by

(2) $$\delta_{y}=\frac{c}{2B}$$

where c is the speed of light. However, previous works approximately evaluate the cross-range resolution $\delta_{x}$ without considering the spatial variant.

Suppose that k=2π/λ represents the wavenumber in free space which is directly related with the wavelength λ. Considering a two-way configuration of 1D-SISO array, which means that the wavenumber $k_{S}=2\cdot k$ corresponding to $PSF_{S}$ is employed [3], the down-range resolution $\delta_{y}$ can be expressed by

(3) $$\delta_{y}=\frac{2\pi }{\mathrm{rang}\_k_{S}}$$

where $\mathrm{rang}\_k_{S}=4\pi B/c$ represents the range of $k_{S}$. Similar to Equation (3), it can be derived that the relationship between the cross-range resolution $\delta_{x}$ and the range of wavenumber at *x*-axis $k_{x}$ as follows

(4) $$\delta_{x}=\frac{2\pi }{\mathrm{range}\_k_{x}}$$

where

(5) $$\mathrm{range}\_k_{x}=k_{S}\left(\sin\theta_{1}+\sin\theta_{2}\right)$$

$\theta_{1}$ and $\theta_{2}$ are given in Figure 1. It can be seen that $\delta_{x}$ varies with the space change of targets.

Then, a simulation is presented to verify the proposed formula of the spatial resolution. For the sake of analyzing the spatial variant of $\delta_{x}$, it is reasonable to assume that the wavelength is a constant. Then, the simulation frequency is assumed to be 33 GHz. The length of the array aperture is 30 cm, with a spatial interval of 3 mm. The distance from the targets to the array in the y direction is 0.5 m.

**Figure 2:**
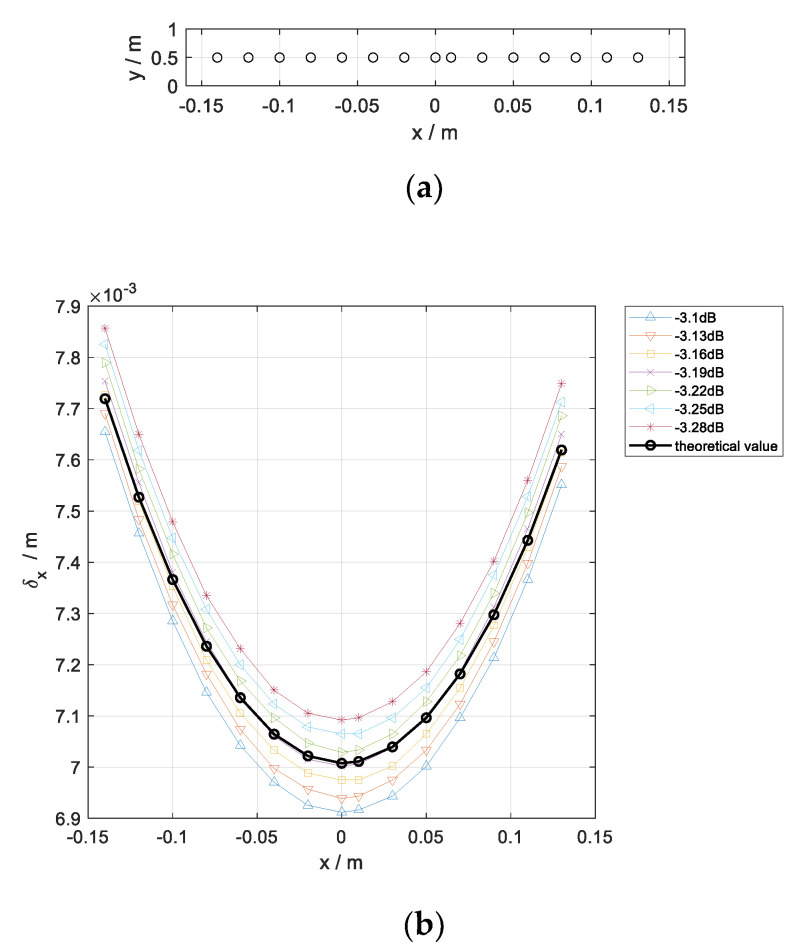
Cross-range resolution simulation diagram; (**a**) Targets distribution; (**b**) Cross-range resolution curve of different magnitude varying with the targets at *x*-axis.

Targets that vary with the *x* coordinate are shown in Figure 2a. Employing the back projection (BP) algorithm, the fitted curves of cross-range resolution varying with the targets are shown in Figure 2b. The black curve represents the theoretical result of cross-range resolution corresponding to Equation (5). Other curves are simulation results at different amplitudes. For comparison purposes, the imaging values are normalized to 0 dB.

In Figure 2b, we can see that the black curve fitted by theoretical values is similar to other curves of simulation values. In addition, the black curve appears between the simulation curve of amplitude –3.19 dB and that of amplitude –3.22 dB. However, the black curve and the two simulation curves do not completely match, and there are a few errors on both sides of the black curve. This phenomenon can be understood as follows: The down-range resolution $\delta_{y}$ is defined as the spread between the two –3 dB points of the echo signal, which is given by a sinc function [14]. Since Equation (4) is derived from Equation (3), the black curve is drawn in terms of the sinc function, while the simulation curves are drawn according to $PSF_{S}$. The $PSF_{S}$ is approximately a sinc function which causes a few errors in the black curve [14]. Since the relative errors between theoretical and simulated values are very small, it is sufficient to evaluate the variation of the cross-range resolution.

As a result, the theoretical resolution is sensitive to the spatial variation of the targets. The main reason for appearing a few errors is that the theoretical values are drawn according to a sinc function, which is not exact match of $PSF_{S}$.

### 2.2. Sampling Criteria

The spatial interval between adjacent transmitter or receiver influences the scope of observation to a large extent. To ensure no aliasing in the observation scope of targets, the Nyquist criteria must be satisfied. It means that the phase sampling interval of complex signals is less than 2π [14]. According to this character, we can obtain the expression of spatial interval,Δx, derived wavenumber domain, as follows:

(6) $$\mathrm{rang}\_k_{D}\cdot \Delta x⩽2\pi$$

where

(7) $$\mathrm{range}\_k_{D}=2k\sin\theta_{D\_half}\sin\theta_{D\_half}=\frac{D/2}{\sqrt{{\left(D/2\right)}^{2}+Y^{2}}}.$$

$\mathrm{range}\_k_{D}$ represents the range of $k_{S}$ corresponding to the distribution range of the targets D. Thus, the spatial interval Δx at the array must satisfy

(8) $$\Delta x⩽\frac{\lambda \sqrt{{\left(D/2\right)}^{2}+Y^{2}}}{2D}.$$

On the other hand, let us assume that the interval of the array Δx is known. When the left side and the right side are equal at Equation (8), the maximum observation range of targets can be determined by

(9) $$D_{\max}=\frac{2\lambda Y}{\sqrt{16\Delta x^{2}-\lambda^{2}}}.$$

It can be seen from Equation (9) that, as the number of the transceivers is few, which means the array interval is large, the scope of observation D is narrow.

As a result, the spatial resolution of a 1D-SISO radar system in near range have been evaluated with the simulation result. The limit of observation range caused by the spatial interval in 1D-SISO array is estimated as well. For $PSF_{T}$ or $PSF_{R}$ where MMW travels in one-way, the results of spatial resolution and sampling criteria can be obtained by replacing $k_{S}$ with k.

## 3. Fast 3D Near Range Imaging Algorithm

**Figure 3:**
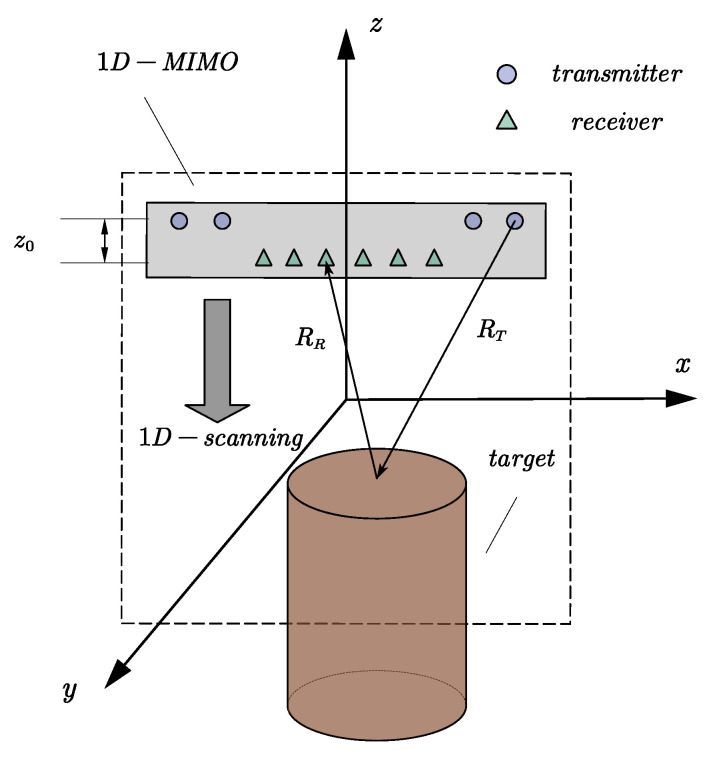
Geometry and coordinate definitions.

The geometry and coordinated definitions of a 1D-scanning-1D-MIMO array and a distributed target are given in Figure 3. The coordinate of the transmitter is $\left(x_{T},0,z\right)$. For practical applications, there is a fixed interval $z_{0}$ between a transmitter array and a receiver array. Thus, the coordinate of the receiver is $\left(x_{T},0,z+z_{0}\right)$. The target’s coordinate is $\left(x^{\prime },y^{\prime },z^{\prime }\right)$. The *x*-axis represents the dimension of the 1D-MIMO array. The *z*-axis represents the dimension of the 1D-scanning. The final result is equivalent to a 1D-scanning-1D-MIMO array.

In order to acquire a three-dimensional image, a linear frequency modulated (LFM) pulse mode is applied. Assuming that the carrier frequency is $f_{c}$. The linear frequency modulation slope is *κ*. Suppose that the scattering process satisfies the Born approximation [15] and ignore the propagation attenuation. If we consider the spatial wavenumber $k=2\pi (f_{c}+kt)/c$ represents time [16,17] we arrive at

(10) $$s\left(k,x_{T},x_{R},z\right)=∭o(x{}^{\prime },y{}^{\prime },z{}^{\prime })\exp\left(-jkR_{T}-jkR_{R}\right)dx{}^{\prime }dy{}^{\prime }dz{}^{\prime }$$

where s(⋅) is the echo signal and o(⋅) represents the reflectivity distribution function. $R_{T}/R_{R}$ which represent the distance from $x_{T}/x_{R}$ to targets satisfy

(11) $$\begin{array}{c} R_{T}=\sqrt{{\left(x{}^{\prime }-x_{T}\right)}^{2}+y{{}^{\prime }}^{2}+{\left(z{}^{\prime }-z-z_{0}\right)}^{2}}\\ R_{R}=\sqrt{{\left(x{}^{\prime }-x_{R}\right)}^{2}+y{{}^{\prime }}^{2}+{\left(z{}^{\prime }-z\right)}^{2}} \end{array}.$$

Corresponding to the BP algorithm is [10]

(12) $$\hat{o}\left(x^{\prime },y^{\prime },z^{\prime }\right)\approx \iint \iint s\left(k,x_{T},x_{R},z\right)\exp\left(jkR_{T}+jkR_{R}\right)dkdx_{T}dx_{R}dz.$$

In the following simulations, the BP algorithm is performed according to the Equation (12).

### 3.1. The Proposed Algorithm

BP algorithm can be applied for arbitrary distributed arrays. However, its computational complexity is tremendous. Therefore, the imaging system cost is raised for affording huge computational quantity [18,19] In addition, current fast algorithms hypothesize that the transmitters or receivers are evenly located [8,10,20]. In practice, such fast algorithms cannot be used if some antennas are damaged. On the other hand, not all application scenarios can locate the arrays evenly. Thus, we extend the evenly distributed of antennas to arbitrary distributed. Firstly, employing the spherical-wave decomposition formula [21,22] which is based on Fourier transform and the principle of stationary phase, we can go further and obtain

(13) $$\begin{array}{c} \exp\left(-jkR_{T}\right)\approx \int \exp\left(-jk_{x_{T},y}\sqrt{{\left(x{}^{\prime }-x_{T}\right)}^{2}+y{{}^{\prime }}^{2}}-jk_{z_{T}}\left(z{}^{\prime }-z-z_{0}\right)\right)dk_{z}\\ \exp\left(-jkR_{R}\right)\approx \int \exp\left(-jk_{x_{R},y}\sqrt{{\left(x{}^{\prime }-x_{R}\right)}^{2}+y{{}^{\prime }}^{2}}-jk_{z_{k}}\left(z{}^{\prime }-z\right)\right)dk_{z} \end{array},$$

where $k_{x_{T},y}$,$k_{x_{R},y}$ stand for wavenumber variables at *x*-*y* plane caused by transmitters or receivers respectively. $k_{z_{T}}$,$k_{z_{R}}$ stand for the similar variables at *z*-axis. Furthermore, $k_{x_{T},y}$,$k_{x_{R},y}$,$k_{z_{T}}$,$k_{z_{R}}$ in Equation (13) should meet the following conditions

(14) $$\begin{array}{c} k_{x_{T},y}^{2}=k^{2}-k_{z_{T}}^{2},k_{x_{T},y}>0\\ k_{x_{R},y}^{2}=k^{2}-k_{z_{R}}^{2},k_{x_{R},y}>0\\ k_{Z_{T}}=k_{Z_{R}}=1/2k_{Z}. \end{array}$$

Due to $k_{z_{T}}=k_{z_{R}}$, we can set $k_{x_{T},y}=k_{x_{R},y}=k_{x,y}$. Substituting (13) and (14) into (10), we are able to derive

(15) $$\begin{array}{ll} s(k,x_{T},x_{R},z)= & \int \int \int \int o\left(x{}^{\prime },y{}^{\prime },z{}^{\prime }\right)\exp\left(-jk_{z}z{}^{\prime }\right)dz{}^{\prime }\exp\left(jk_{z}\left(z+\frac{1}{2}z_{0}\right)\right)dk_{z}\\ & \exp\left(-jk_{x,y}\left(\sqrt{{\left(x{}^{\prime }-x_{T}\right)}^{2}+y{{}^{\prime }}^{2}}+\sqrt{{\left(x{}^{\prime }-x_{R}\right)}^{2}+y{{}^{\prime }}^{2}}\right)\right)dx{}^{\prime }dy{}^{\prime }. \end{array}$$

Then, apply Fourier transform on both sides with respect to z and $z^{\prime }$ at (15), we can obtain

(16) $$\begin{array}{ll} s\left(k,x_{T},x_{R},k_{z}\right) & ={\mathrm{FT}}_{z}\left[s\left(k,x_{T},x_{R},z\right)\right]\\ & =\iint {\mathrm{FT}}_{z{}^{\prime }}\left[o\left(x{}^{\prime },y{}^{\prime },z{}^{\prime }\right)\right]\exp\left(-jk_{x,y}\left(\sqrt{{\left(x{}^{\prime }-x_{T}\right)}^{2}+y{{}^{\prime }}^{2}}+\sqrt{{\left(x{}^{\prime }-x_{R}\right)}^{2}+y{{}^{\prime }}^{2}}\right)\right)dx{}^{\prime }dy{}^{\prime }\\ & =\iint o\left(x{}^{\prime },y{}^{\prime },k_{z}\right)\exp\left(-jk_{x,y}\left(\sqrt{{\left(x{}^{\prime }-x_{T}\right)}^{2}+y{{}^{\prime }}^{2}}+\sqrt{{\left(x{}^{\prime }-x_{R}\right)}^{2}+y{{}^{\prime }}^{2}}\right)\right)dx{}^{\prime }dy{}^{\prime } \end{array}$$

There is a Fourier transform pair

(17) $$z+\frac{1}{2}z_{0}\to k_{z}.$$

Then, we transform the 4D data of echo signal $s\left(k,x_{T},x_{R},k_{z}\right)$ into a 3D data $O\left(k_{x,y},x_{T},x_{R}\right)$ constrained by Equation (14). It can be seen that Equation (16) is similar to Equation (10). Thus, employing BP algorithm to deal Equation (16) at *x-y* plane, we can obtain

(18) $$\begin{array}{ll} o\left(x{}^{\prime },y{}^{\prime },k_{z}\right)= & ∭O\left(k_{x,y},x_{T},x_{R}\right)\\ & \exp\left(jk_{x,y}\left(\sqrt{{\left(x{}^{\prime }-x_{T}\right)}^{2}+y{{}^{\prime }}^{2}}+\sqrt{{\left(x{}^{\prime }-x_{R}\right)}^{2}+y{{}^{\prime }}^{2}}\right)\right)dx_{T}dx_{R}dk_{x,y} \end{array}$$

Finally, we can obtain the imaging equation as follows

(19) $$\hat{o}\left(x{}^{\prime },y{}^{\prime },z{}^{\prime }+\frac{1}{2}z_{0}\right)=IFT_{k_{z}}\left(o\left(x{}^{\prime },y{}^{\prime },k_{z}\right)\right).$$

**Figure 4:**
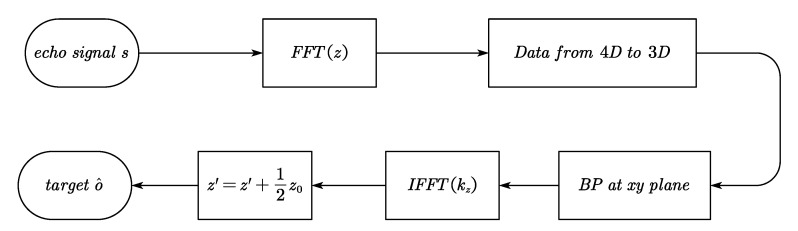
The main steps flow of the proposed algorithm.

To apply the fast Fourier transform (FFT) with respect to *z* in Equation (16) or the inverse fast Fourier transform (IFFT) with respect to $k_{z}$ in Equation (19), the 1D-scanning interval is considered to be even, which is consistent with actual applications. Since Equation (13) is derived from the spherical-wave decomposition equation, which is usually suitable for near range imaging applications [20,22], the proposed algorithm is a good trade-off between efficiency and accuracy for arbitrarily distributed 1D-MIMO array. The main steps of the proposed algorithm are summarized in Figure 4.

### 3.2. Imaging Quality

**Figure 5:**
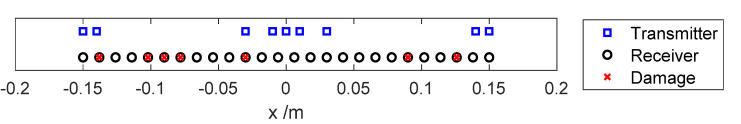
Array structure for simulation.

In this section, imaging quality of different algorithms is compared by qualitative and quantitative analysis. Firstly, the MIMO array structure is shown in Figure 5, whose receivers are evenly distributed. The location selection of transmitting antennas is based on experimental experience, and the optimization of them is not involved in this paper. Then, the array moves equidistant along the *z* direction. A fast algorithm is named enhanced algorithm, for such an array whose receivers are evenly distributed has been proposed in [10].In order to verify the flexibility of the proposed algorithm, we suppose that a few receivers are damaged in the array to achieve sparse distribution. In the following process, the original array is called ARRAY A, and the damaged array is called ARRAY B.

Detailed simulation parameters are listed in Table 1, $L_{xT}$, $L_{xR}$ represent the aperture length of transmitter array and that of the receiver array respectively. Furthermore, $L_{z}$ represents synthetic aperture length in the direction of mechanical scanning. Moreover, Δz is the scanning interval.

**Figure 6:**
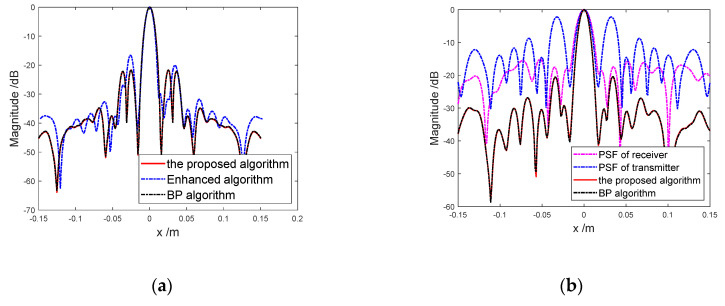
PSFs of different algorithms: (**a**) PSFs of different algorithms for ARRAY A; (**b**) PSFs of receiver, transmitter and different algorithms for ARRAY B.

Then, suppose an ideal unit scattering point is located at (0,0.5,0). In image processing, the 3D results are firstly sliced to the *x*-*z* plane at *y* = 0.5m. Then, the PSFs along the array direction at *z* = 0m are plotted in Figure 6.

For ARRAY A, PSFs of enhanced algorithm, the proposed algorithm and BP algorithm are shown in Figure 6a. Intuitively, it can be seen that $PSF_{M}$ of the proposed method is superior to that of the enhanced algorithm. After a few of the receivers are damaged, PSFs of the proposed algorithm and BP algorithm are shown in Figure 6b. Meanwhile, Figure 6b shows $PSF_{T}$ and $PSF_{R}$ respectively, which are plotted by the way of BP algorithm. It is obvious that $PSF_{M}$ of BP algorithm is the product of $PSF_{T}$ and $PSF_{R}$, which verify the correctness of Equation (1). Meanwhile, it can be seen that $PSF_{M}$ is mainly determined by $PSF_{R}$ due to the few transmit antennas. In Figure 6a,b, we can also see that $PSF_{M}$ of BP algorithm are approximately consistent with that of the proposed algorithm. It means that the image quality of the proposed algorithm almost achieves the best standard accuracy.

As quantitative values, the peak side-lobe ratio (PSLR) and the integrated side lobe ratio (ISLR) are used to evaluate the image quality of different algorithms. Table 2 lists the PSLRs and ISLRs corresponding to Figure 6a,b.

In Table 2, both indexes of the proposed algorithm tend to them of BP algorithm, and better than them of enhanced algorithm for ARRAY A. For ARRAY B, both indexes of the two algorithms are close to each other. Only judging from the indexes, we can conclude that the proposed algorithm obtains the high imaging accuracy, which is consistent with that of BP algorithm.

### 3.3. Computational Complexity

As can be seen from the above proof procedure, the proposed algorithm can use the fast Fourier transform at *z*-axis, compared with the BP algorithm. In order to obtain a more accurate estimation, let $N_{k}$ represent the number of frequency points, $N_{z}$ represent the number mechanical scanning samples. $N_{x_{T}}$ and $N_{x_{R}}$ represent the number of transmitters and receivers respectively. Suppose that the number of points in each direction of *x*-axis, *y*-axis, *z*-axis is $N_{x^{\prime }}$, $N_{y^{\prime }}$, $N_{z^{\prime }}$ respectively. Then, let α represent the computation cost for one-dimensional FFT or IFFT operation, and β represent the computation cost for one pixel point projection from a pair of transmitter and receiver at *x*-*y* plane. We can obtain calculated quantities corresponding to the main steps of the proposed algorithm from Table 3.

Suppose that $N_{k}$, $N_{x_{T}}$, $N_{x_{R}}$, $N_{z}$ and $N_{x^{\prime }}$, $N_{y^{\prime }}$, $N_{z^{\prime }}$ are in the same order N. Consequently, the computational complexity of BP algorithm, enhanced algorithm and the proposed algorithm are $O\left(N^{7}\right)$, $O\left(N^{5}\log N\right)$, $O\left(N^{6}\right)$ respectively. It can be seen that the computational complexity of the proposed algorithm is less than that of BP algorithm. Although enhanced algorithm takes less time cost, its array location is limited. In the following simulation, the advantage of the proposed algorithm about the time cost and flexibility will be verified with numerical simulation results and experimental results.

## 4. Experimental Results

### 4.1. Numerical Simulations

To validate the proposed algorithm, numerical simulations are carried out in this section. The commercial electromagnetic computation software FEKO (Altair Engineering, Troy, MI, USA) is used for setting a representative geometric model and electromagnetic (EM) calculation, finally, given the comparison results of the proposed algorithm, enhanced algorithm and the BP algorithm.

**Figure 7:**
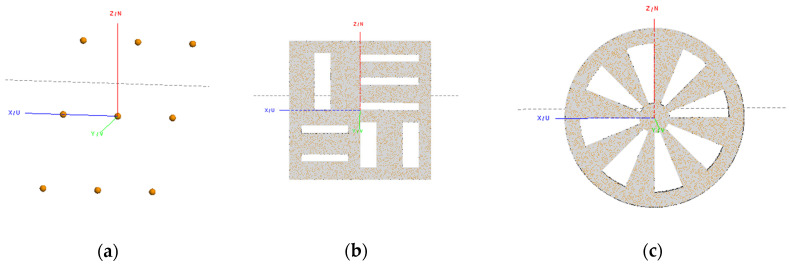
(**a**) CAD model of nine identical spheres by FEKO; (**b**) CAD model of a slab square after meshing by FEKO; (**c**) CAD model of a Siemens Star after meshing by FEKO.

In the electromagnetic numerical simulation, FEKO is used for computer-aided design (CAD) modeling and EM calculating. The established CAD models are drawn in Figure 7. In Figure 7a, nine identical spheres that are extremely small are used as ideal scatter point targets. Distributed targets, including a slab square and a Siemens Star, are further used for testing the imaging performance in Figure 7b,c. The slab square is sensitive to the horizontal and vertical cross-range resolution. The Siemens Star is used to comprehensively evaluate the imaging quality in all directions. The three models are set by default, as follows:

- The spheres are located in the coordinate (−0.05, 0.6, 0.05), (0, 0.6, 0.05), (0.05, 0.6, 0.05), (−0.05, 0.5, 0), (0, 0.5, 0), (0.05, 0.5, 0), (−0.05, 0.4, −0.05), (0, 0.4, −0.05), (0.05, 0.4, −0.05) and the radii of them are 0.003 m (about a third of the center wavelength).
- The outer ring radius, inner ring radius and thickness of Siemens Star are 0.06 m, 0.01 m and 0.002 m, respectively.
- The horizontal, vertical interval and side-length of slab square are 0.007m, 0.0014m and 0.12m, respectively.
- All models are assumed to be perfect electric conductors.
- All models are meshed with physical optics (PO)-full ray-tracing.
- An electric dipole is used as a transmitter.
- A near-field point is designed as a receiver and is calculated only at the scattered part of the field.

After one excitation, the echo waves at all triangular patches of the target were solved by FEKO calculation and a group of data were obtained. The above process was repeated with updated transmitter and receiver positions. Finally, the required 1D-MIMO-1D-scanning data were obtained.

According to the analysis in Section 2, three simulations are carried out firstly to analyze point target response and verify resolution requirements and spatial sampling criteria.

The image parameters are set equal to that of Table 1. The 1D-MIMO array is also displayed as ARRAY A. The resolution in the *z* direction can be calculated as 0.007 m from the Equation (4), and the resolution of the receiver array in the *x* direction is 0.014 m. The reason for only considering the receiver array is that there are few transmitters, which have little effect on the $PSF_{M}$.

**Figure 8:**
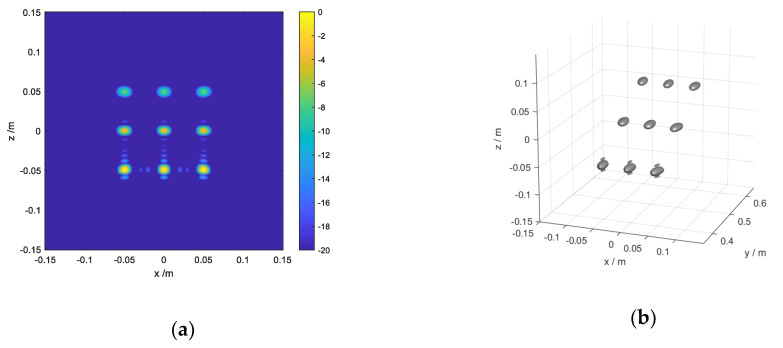
Imaging results of nine identical spheres: (**a**) 2D imaging result; (**b**) 3D imaging result.

Firstly, the response results of point targets are shown in Figure 8 in the form of a 2D imaging result and a 3D imaging result. The nine points whose color tend to be blue must be farther than the others in Figure 8a. Additionally, it can be seen obviously that the top three points are farthest to the scanning plane, whose *y*-coordinate component is 0.6 m in Figure 8b. Thus, we can conclude that the position of point targets is presented accurately with the figure function.

**Figure 9:**
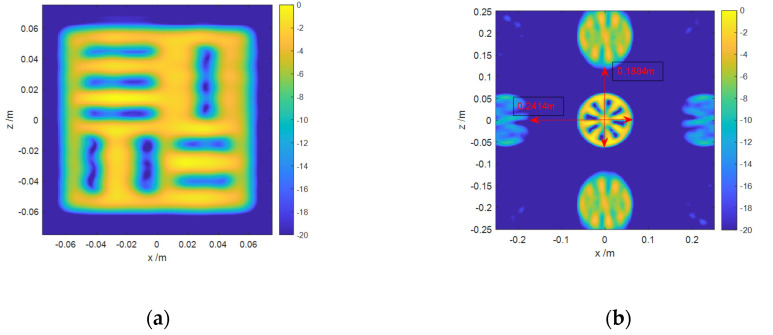
Imaging results of different models: (**a**) 2D imaging result of a slab square; (**b**) 2D imaging result of a Siemens Star.

Secondly, the 2D imaging results of the slab square are shown in Figure 9a. Whether in the *x* direction or the *z* direction, the gap in the middle is obviously larger than that on both sides, which proves the spatial variant of the cross-range resolution.

Thirdly, it is assumed that the scanning interval Δz is 0.012 m, and the receiver array interval is 0.02 m. From the Equation (9), the observation range in the z direction can be calculated as 0.2333 m, and the observation range in the *x* direction is 0.1928 m. All the other parameters are consistent with the point targets simulation. The 2D imaging result of the Siemens Star is shown in Figure 9b. It can be seen intuitively that the blur range in the *x* direction and the *z* direction is 0.1884 m and 0.2414 m, respectively. The result is similar to the theoretical result, which proves the rationality of Equation (9) as well.

**Figure 10:**
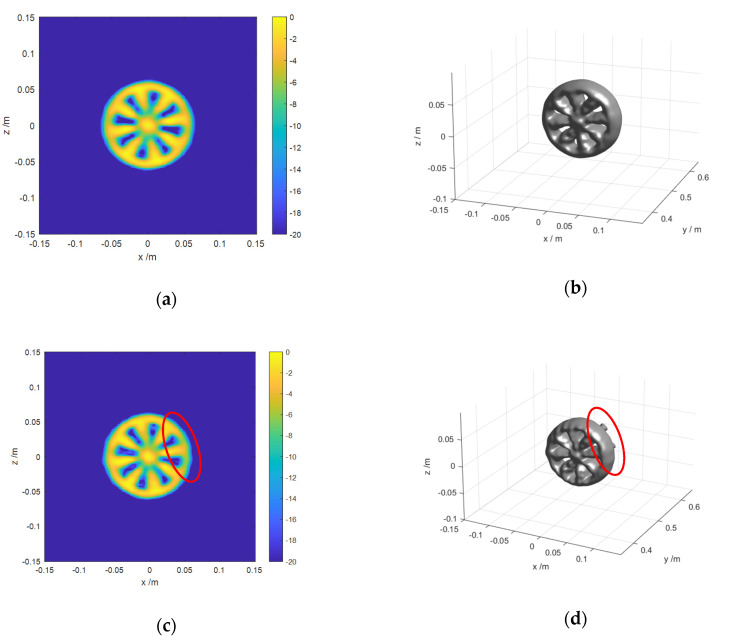
Imaging results of different algorithms for ARRAY A: (**a**,**b**) are the imaging result of the proposed algorithm; (**c**,**b**) are the imaging result of Enhanced algorithm.

**Figure 11:**
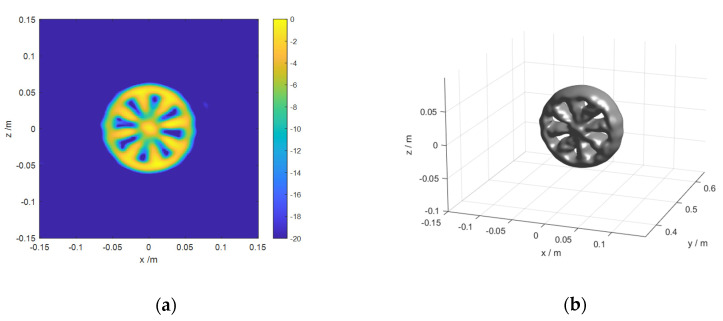

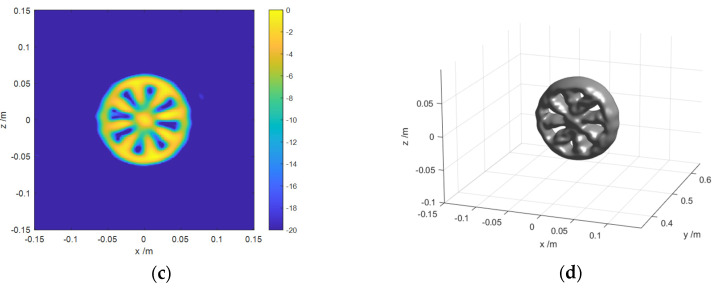
Imaging results of different algorithms for ARRAY B: (**a**,**b**) are the imaging result of the proposed algorithm; (**c**,**d**) are the imaging result of BP algorithm.

Then, a series of simulations are carried out to verify the feasibility of the proposed algorithm, by comparing it with other algorithms. The image parameters are consistent with those in Table 1. Figure 10 shows the imaging results of the proposed algorithm and enhanced algorithm in the case of 256 × 15 × 256 voxels. Moreover, 2D imaging results are the maximum projection of the 3D imaging results on the *x-z* plane. The values of all voxels are normalized to 0 dB. The dynamic range of 2D imaging results is 20 dB. The magnitude of 3D imaging surface is 15 dB. As can be seen, Figure 10c,d show defocusing and aliasing slightly at the right side compared with Figure 10a,b. It indicates that the imaging quality of the enhanced algorithm is worse than that of the proposed one. For ARRAY B, the 2D and 3D imaging results of the proposed algorithm and BP algorithm are shown in Figure 11.

Figure 11 shows the imaging results of the proposed algorithm and BP algorithm. Comparing a,b and c,d in Figure 11, we can see intuitively that the imaging results obtained by the proposed algorithm and BP algorithm are of high consistency. the results verify the effectiveness of the proposed algorithms. However, the imaging time of the both algorithms varies greatly. Detailed time cost is shown in Table 4.

The time cost of the three algorithms for different arrays is listed in Table 4. The imaging results are simulated by the same PC with Intel(R) core (TM) i7-9750H CPU (Intel Corporation, Santa Clara, CA, USA), using MATLAB (R2019a, MathWorks, Natick, MA, USA), and no parallel computing technique is used. It can be seen that the time cost of enhanced algorithm, the proposed algorithm and BP algorithm increases successively. The reason can be summarized as follows: For Enhanced algorithm, FFT are applied to two dimensions of transmitters and 1D-scanning. On the contrary, FFT is only applied to 1D-scanning for the proposed algorithm. Although enhanced algorithm needs less time than the proposed one, its imaging results are not better than that of the proposed algorithm under ARRAY A. In addition, the data process of the proposed algorithm takes much less time than that of the BP algorithm. It verifies the superiority of the proposed algorithm on computational calculation, and validates the time-cost result of theoretical derivation as well.

### 4.2. Experimental Simulations

**Figure 12:**
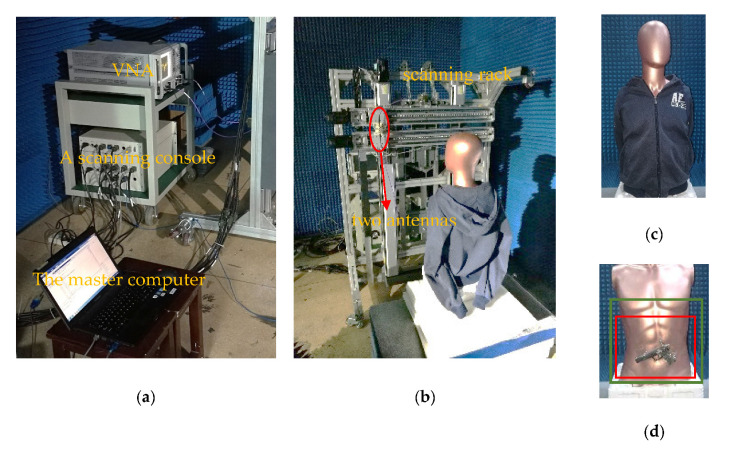
The experimental scenario: (**a**) Photograph of a vector network, a console and a computer; (**b**) Photograph of a customized scanning rack; (**c**) Photograph of a man model with a coat; (**d**) Photograph of a man model tied to a gun.

**Figure 13:**
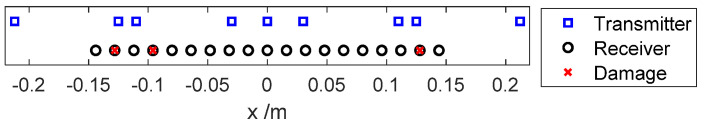
Array structure for experiment.

The main instruments used in the experiments consist of a computer, a vector network analyzer (VNA), a scanning console, a customized scanning rack and a couple of antennas. VNA is shown in Figure 12a as a signal source. The console connects the computer and the scanning rack for linkage control. A customized scanning machine is shown in Figure 12b. There are three spindles that can be controlled separately. The horizontal two spindles control transmit antenna and receive antenna respectively, and the other one carries out 1D-scanning. Thus, an equivalent 1D-scanning-1D-MIMO regime can be achieved. Two same horn antennas have an approximate 40° beamwidth, which should be considered when designing 1D-MIMO array configuration. Set Port2 of VNA to transmit signal and port1 to receive signal. the target is shown in Figure 12c,d. We tie a gun model to a man model and put a coat on it. In Figure 12d, the area bounded by a red rectangle is the scanning area of 1D-MIMO array and the area bounded by a green rectangle is the imaging area. The distance between the target and the scanning rack is 0.5 m. The 1D-sacnning ranges from –0.15 to 0.15 m, sampling a total of 51 points. The following array structure is used to illuminate the target completely shown in Figure 13. The MIMO array before damage is named ARRAY C and damaged array named ARRAY D. For ARRAY C, the observation range in the *z* direction can be calculated as 0.6765 m and the observation range in the *x* direction is 0.2962 m from Equation (9).

**Figure 14:**
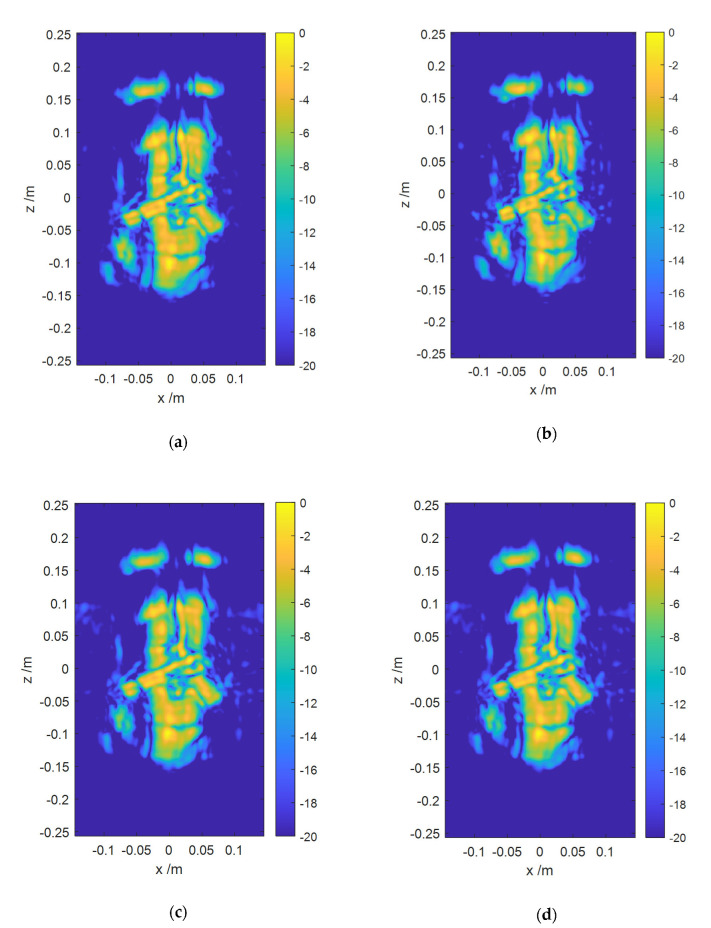
Imaging results of the man model with a coat: (**a**) Imaging results of the proposed algorithm for ARRAY C; (**b**) Imaging results of Enhanced algorithm for ARRAY C; (**c**) Imaging results of the proposed algorithm for ARRAY D; (**d**) Imaging results of BP algorithm for ARRAY D.

In Figure 14a,b, the displayed dynamic range is 20 dB, and the concealed gun model imaged by the proposed and BP algorithm can be clearly observed. However, the background noise has emerged in Figure 14b, employing the enhanced algorithm. In Figure 14c,d, the imaging results of the proposed algorithm and BP algorithm are similar to each other. It means that the proposed algorithm is highly consistent with the imaging results obtained by the BP algorithm. Therefore, we can conclude that the proposed algorithm achieves a complete focus on the target. The reason for the background noise on both sides in Figure 14c,d is that the observation range calculated at *x*-axis above is no longer valid with damage of the receiver’s array, which results in the appearance of the side lobe above 20 dB in the observed area.

The time cost of different algorithms is shown in Table 5. Although the enhanced algorithm takes less time than the proposed algorithm, it requires uniform distribution of the receiver array, and the visual quality of the image is no better than that of the proposed algorithm. Meanwhile, the time cost of the proposed algorithm is no more than 1% the time cost of the BP algorithm for both arrays. On the other hand, Since the array position of the proposed method is not limited, transmitters and receivers can be distributed according to the actual situation. Considering the analysis about the relationship between the array position and the imaging performance in Section 2, The 1D-scannig-1D-MIMO array can be optimized according to the imaging target region and required resolution.

## 5. Conclusions

In this paper, we propose an efficient and accurate three-dimension imaging algorithm for 1D-scanning-1D-MIMO array in near range applications. The proposed algorithm is applicable to the case where transmitters and receivers are both arbitrarily distributed. Detailed theoretical derivations and specific algorithm steps are presented as well. In array design section, the spatial resolution is discussed though, considering the spatial variability of cross-range resolution. Due to observation scene, the array interval is also investigated. Moreover, they are verified by numerical simulation results. In the simulation and experiment sections, the image performance and computational efficiency of the proposed algorithm are demonstrated both with numerical simulations and measurements of distributed targets. Meanwhile, the proposed algorithm is compared with classical BP algorithm and fresh enhanced algorithm. Results show that, in the cases where enhanced algorithm is applied, the proposed method has better imaging quality; and in the cases where enhanced algorithm is no longer valid, the proposed algorithms can achieve fully focused imaging while taking less than 1% the time needed for the BP algorithm. In practice, if individual antennas are damaged or there are application scenarios where the array cannot be located evenly, the proposed algorithm opens up a possibility to develop a high-quality real-time imaging system by using 1D-scannning-1D-MIMO arrays and powerful parallel computers. As a result, it indicates that the proposed algorithm will accelerate the progress of 1D-MIMO-1D-scanning system in practical applications.

## Figures and Tables

**Table 1:** Parameters configuration.

*f_c_*	*B*	$L_{z}$	*L_xR_*	Δz
33 GHz	6 GHz	0.3 m	0.3 m	0.006 m

**Table 2:** Imaging performance of different algorithms.

Algorithm	PLSR (dB)	ISLR (dB)
The proposed algorithm (A)	−21.6209	−17.4170
Enhanced algorithm (A)	−16.5958	−13.7017
BP algorithm (A)	−21.6414	−17.3811
The proposed algorithm (B)	−20.5030	−16.1217
BP algorithm (B)	–20.4073	–16.0615

**Table 3:** Computation complexity for main steps of the proposed algorithm.

The Main Operating	Calculated Quantities
FFT(z)	$\alpha N_{k}N_{x,T}N_{x,R}N_{k_{z}}\log_{2}N_{k_{z}}$
BP at x−y plane	$\beta N_{k}N_{k_{z}}N_{x,T}N_{x,R}N_{x^{\prime }}N_{y^{\prime }}$
$\mathrm{IFFT}\left(k_{z}\right)$	$\alpha N_{k}N_{x,T}N_{x,R}N_{z}\log_{2}N_{z}$

**Table 4:** Comparison on time cost of different algorithms for numerical simulation.

Algorithm	Time Cost
The proposed algorithm (A)	53.4809 s
Enhanced algorithm (A)	6.4273 s
The proposed algorithm (B)	39.4707 s
BP algorithm (B)	2820.4890 s

**Table 5:** Comparison on time cost of different algorithms for experimental simulation.

Algorithm	Time Cost
The proposed algorithm (C)	270.6647 s
Enhanced algorithm (C)	45.6625 s
The proposed algorithm (D)	228.3791 s
BP algorithm (D)	25075.1153 s

## References

[1] Liu H., Zhang Y.X., Long Z.J., Han F., Liu Q.H. Three-dimensional reverse-time migration applied to a MIMO GPR system for subsurface imaging. Proceedings of the 2016 16th International Conference on Ground Penetrating Radar (GPR).

[2] Zhuge X., Yarovoy A.G. (2012). Study on Two-Dimensional Sparse MIMO UWB Arrays for High Resolution Near-Field Imaging. IEEE Trans. Antennas Propag..

[3] Sheen D.M., McMakin D.L., Hall T.E. (2001). Three-dimensional millimeter-wave imaging for concealed weapon detection. IEEE Trans. Microw. Theory Tech..

[4] Steinberg B.D. (1976). Principles of Aperture and Array Systems Principles of Aperture and Array Systems Design.

[5] Qiao L., Wang Y., Zhao Z., Chen Z. (2015). Exact Reconstruction for Near-Field Three-Dimensional Planar Millimeter-Wave Holographic Imaging. J. Infrared Millim. Terahertz Waves.

[6] Zhuge X., Yarovoy A.G. (2011). A Sparse Aperture MIMO-SAR-Based UWB Imaging System for Concealed Weapon Detection. IEEE Trans. Geosci. Remote Sens..

[7] Sheen D.M. Sparse Multi-static Arrays for Near-field Millimeter-wave Imaging. Proceedings of the 2013 IEEE Global Conference on Signal and Information Processing.

[8] Gumbmann F., Schmidt L. (2011). Millimeter-Wave Imaging with Optimized Sparse Periodic Array for Short-Range Applications. IEEE Trans. Geosci. Remote Sens..

[9] Baccouche B., Agostini P., Mohammadzadeh S., Kahl M., Weisenstein C., Jonuscheit J., Keil A., Löffler T., Sauer-Greff W., Urbansky R. (2017). Three-Dimensional Terahertz Imaging With Sparse Multistatic Line Arrays. IEEE J. Sel. Top. Quantum Electron..

[10] Gao J., Qin Y., Deng B., Wang H., Li X. (2018). Novel Efficient 3D Short-Range Imaging Algorithms for a Scanning 1D-MIMO Array. IEEE Trans. Image Process..

[11] Roberts W., Stoica P., Li J., Yardibi T., Sadjadi F.A. (2010). Iterative Adaptive Approaches to MIMO Radar Imaging. IEEE J. Sel. Top. Signal Process..

[12] Tan X., Roberts W., Li J., Stoica P. (2011). Sparse Learning via Iterative Minimization with Application to MIMO Radar Imaging. IEEE Trans. Signal Process..

[13] Naidu P.S. (2009). Sensor Array Signal Processing.

[14] Cumming I.G., Wong F.H. (2005). Digital Signal Processing of Synthetic Aperture Radar Data: Algorithms and Implementation.

[15] Gubernatis J.E., Domany E., Krumhansl J.A. (1977). The Born approximation in the theory of the scattering of elastic waves by flaws. J. Appl. Phys..

[16] Lopez-Sanchez J.M., Fortuny-Guasch J. (2000). 3-D radar imaging using range migration techniques. IEEE Trans. Antennas Propag..

[17] Guo Q., Zhang X., Chang T., Cui H.-L., Tian X. (2017). Three-dimensional bistatic array imaging using range migration algorithm. Electron. Lett..

[18] Ahmed S.S., Schiessl A., Schmidt L. (2011). A Novel Fully Electronic Active Real-Time Imager Based on a Planar Multistatic Sparse Array. IEEE Trans. Microw. Theory Tech..

[19] Ahmed S.S., Schiessl A., Gumbmann F., Tiebout M., Methfessel S., Schmidt L. (2012). Advanced Microwave Imaging. IEEE Microw. Mag..

[20] Zhuge X., Yarovoy A.G. (2011). Sparse multiple-input multiple-output arrays for high-resolution near-field ultra-wideband imaging. IET Microw. Antennas Propag..

[21] Zhang B., Pi Y., Min R. (2013). A near-field 3D circular Sar imaging technique based on spherical wave decomposition. Prog. Electromagn. Res..

[22] Gao J., Qin Y., Deng B., Wang H., Li X. (2018). A Novel Method for 3-D Millimeter-Wave Holographic Reconstruction Based on Frequency Interferometry Techniques. IEEE Trans. Microw. Theory Tech..

